# Association between the extent of house collapse and urine sodium-to-potassium ratio of victims affected by the 2011 Great East Japan Earthquake and Tsunami: a cross-sectional study

**DOI:** 10.1038/s41440-023-01190-5

**Published:** 2023-02-17

**Authors:** Takahiro Mikami, Kozo Tanno, Ryohei Sasaki, Nobuyuki Takanashi, Yuka Kotozaki, Koichi Asahi, Fumitaka Tanaka, Shinichi Omama, Mana Kogure, Naoki Nakaya, Tomohiro Nakamura, Naho Tsuchiya, Akira Narita, Atsushi Hozawa, Jiro Hitomi, Kiyomi Sakata, Makoto Sasaki

**Affiliations:** 1 Division of Clinical Research and Epidemiology, Iwate Tohoku Medical Megabank Organization, Iwate Medical University, 1-1-1 Idaidori, Yahaba-cho, Shiwa-gun, Iwate 028-3694 Japan; 2 Department of Anatomy, School of Medicine, Iwate Medical University, 1-1-1 Idaidori, Yahaba-cho, Shiwa-gun, Iwate 028-3694 Japan; 3 Department of Hygiene and Preventive Medicine, School of Medicine, Iwate Medical University, 1-1-1 Idaidori, Yahaba-cho, Shiwa-gun, Iwate 028-3694 Japan; 4 Department of Human Sciences, Center for Liberal Arts and Sciences, Iwate Medical University, 1-1-1 Idaidori, Yahaba-cho, Shiwa-gun, Iwate 028-3694 Japan; 5 Department of Internal Medicine, School of Medicine, Iwate Medical University, 1-1-1 Idaidori, Yahaba-cho, Shiwa-gun, Iwate 028-3694 Japan; 6 Iwate Prefectural Advanced Critical Care and Emergency Center, Iwate Medical University, 1-1-1 Idaidori, Yahaba-cho, Shiwa-gun, Iwate 028-3694 Japan; 7 Tohoku Medical Megabank Organization, Tohoku University, 2-1 Seiryo-machi, Aoba-ku, Sendai-City, Miyagi Japan; 8 Institute for Biomedical Sciences, Iwate Medical University, 1-1-1 Idaidori, Yahaba-cho, Shiwa-gun, Iwate 028-3694 Japan

**Keywords:** Natural disasters, Potassium, Sodium, Sodium-to-potassium ratio, Urine

## Abstract

People who experience natural disasters have a high risk of developing cardiovascular diseases. We investigated the association between the extent of house collapse and urine sodium-to-potassium (UNa/K) ratio of 2011 Great East Japan Earthquake victims. We used the baseline survey data of the Tohoku Medical Megabank Project Community-Based Cohort Study of 29 542 individuals (aged 20–74 years) residing in the affected areas. The UNa/K ratio was calculated using spot urinary electrolyte values. Analysis of covariance was used to calculate the multivariate-adjusted geometric means of the UNa/K ratio in the following groups stratified according to the self-reported extent of house collapse: total collapse (TC), half collapse (HC), partial collapse (PC), and no damage (ND). Multivariable-adjusted odds ratios (ORs) for a high UNa/K ratio were calculated using logistic regression. The TC, HC, PC, and ND groups comprised 5 359 (18.1%), 3 576 (12.1%), 7 331 (24.8%), and 13 276 (44.9%) participants, respectively. The TC (3.33; 95% confidence interval [CI], 3.28–3.38), HC (3.37; 3.30–3.43), and PC (3.32; 3.28–3.37) groups had significantly higher multivariate-adjusted geometric means of the UNa/K ratio than the ND (3.24; 3.21–3.27) group. The multivariable-adjusted ORs (95% CIs) for a high UNa/K ratio in the TC, HC, and PC groups *vs*. the ND group were 1.07 (0.99–1.15), 1.20 (1.11–1.31), and 1.20 (1.12–1.28), respectively. Similar associations between house collapse and UNa/K ratio were observed for both sexes. We report that victims of a natural disaster tend to have a diet with high sodium-to-potassium ratio.

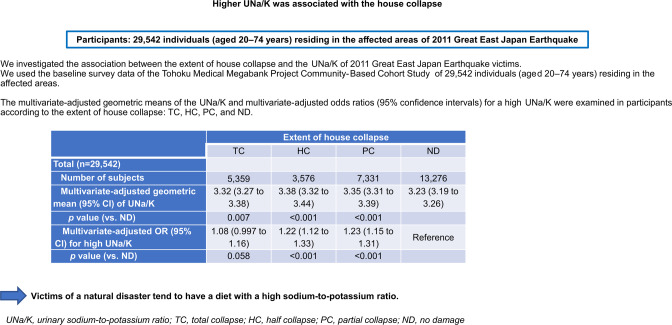

## Introduction

People who experience a natural disaster are at a higher risk of developing cardiovascular disease (CVD) in the acute phase [1–7]. However, higher CVD incidence and mortality rates were also observed during the chronic phase after a disaster [8, 9]. “Acute phase” and “chronic phase” refer to the period within 3 months and >3 years after the disaster, respectively. After the 2011 Great East Japan Earthquake (GEJE), a high incidence of CVDs, such as stroke [10], heart failure [11, 12], and acute myocardial infarction [12, 13], was observed in the acute phase. The incidence of stroke [10, 14] or heart failure [12] was particularly high in areas that were badly flooded by the tsunami due to the 2011 GEJE, and the incidence of acute myocardial infarction positively correlated with the seismic scale of the earthquake [12]. Furthermore, the increase in the incidence of fatal myocardial infarction [15] or acute decompensated heart failure [16] was sustained 3 years after the 2011 GEJE.

Excessive sodium intake [17, 18] and insufficient potassium intake [18] are important risk factors for hypertension and CVD. Recent epidemiological studies conducted not only in the Japanese population but also in multi-ethnic general populations support the usefulness of the urinary sodium-to-potassium (UNa/K) ratio as a predictor of hypertension risk [19] or CVD risk [20]. Their findings suggested that the UNa/K ratio may be superior to using individual urinary sodium or potassium level as an indicator of blood pressure level, not only in the Japanese population [21] but also in multi-ethnic general populations [22]. Since the occurrence of the 2011 GEJE, disaster victims have tended to consume an unbalanced diet [23, 24]. Nutritional imbalances may be observed among long-term evacuees and may be one of the causes of high CVD incidence during the chronic phase in victims affected by a disaster. However, to our knowledge, no studies have focused on the sodium or potassium intake of disaster victims, including those of the 2011 GEJE.

We hypothesized that the victims of a large-scale natural disaster have an unbalanced diet, which leads to a higher intake of salted food and a lower intake of fruits and vegetables that are rich in potassium. This study focused on the extent of house collapse as an indicator of disaster damage and aimed to investigate the association between the extent of house collapse and UNa/K ratio among the victims of the 2011 GEJE.

## Methods

This study was reported using the STROBE guidelines [25].

### Study population

In Japan, both the GEJE and tsunami of March 11, 2011, caused unprecedented damage to the Pacific coast of Iwate and Miyagi Prefectures, which are located in northeastern Japan (Supplementary Figs. 1 and 2). More than 120,000 buildings, which included various facilities used for commercial, educational, and business purposes, were completely destroyed, 278,000 were half-destroyed, and 726,000 were partially destroyed.

To investigate the health conditions of community residents in the areas affected by the 2011 GEJE, the Tohoku University Tohoku Medical Megabank Organization (ToMMo) and Iwate Medical University Iwate Tohoku Medical Megabank Organization launched the Tohoku Medical Megabank (TMM) Project in 2011 [26, 27]. One of the components of this project was the Tohoku Medical Megabank Project Community-Based Cohort Study (TMM CommCohort Study), a prospective cohort study. In this cohort study, 97 419 individuals were requested to visit municipality-specific health check-up sites between May 2013 and March 2016. Among them, 67 355 participants aged 20–74 years were enrolled (agreement rate: 69.1%). The recruitment methodology has been previously described [26, 27].

**Fig. 1:**
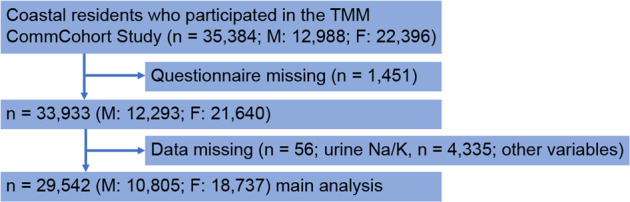
Flowchart of the participant inclusion process

To investigate the association between the extent of house collapse and UNa/K ratio, we focused on the data of 35,384 participants (12,988 men and 22,396 women) living in the coastal areas of Iwate and Miyagi Prefectures. Among them, 5842 were excluded for the following reasons: 1451 did not respond to the questionnaire, and 4391 had missing data on urinary electrolyte levels (*n* = 56) or other important variables (*n* = 4335) (Fig. 1). Finally, the complete data of 29,542 participants (10,805 men and 18,737 women) who were living in the coastal areas were analyzed in this study. Furthermore, we analyzed the complete data of 21,495 coastal residents (7125 men and 14,370 women) who were not receiving antihypertensive treatment because antihypertensive drugs have some effect on urine sodium or potassium excretion.

All study procedures were approved by the Ethics Committee of ToMMo (first approval: 2012-4-617; latest approval: 2018-4-087) and the Medical Ethics Committee of Iwate Medical University (HG H25-2). Written informed consent was obtained from all participants.

### Extent of house collapse

The extent of house collapse was assessed according to one of the six responses to a question about house damage: “A: Completely destroyed,” “B: Half destroyed (requires major repairs),” “C: Half destroyed (requires minor repairs),” “D: Partially destroyed,” “E: No damage,” and “F: Was not living in the affected area.” Respondents who answered A were classified into the total collapse (TC) group, those who answered B or C into the half collapse (HC) group, those who answered D into the partial collapse (PC) group, and those who answered E or F into the no damage (ND) group.

### Calculation of the UNa/K ratio

The UNa/K ratio was calculated using spot urinary electrolyte values. Spot urine specimens were obtained when participants visited municipal-specific health check-up sites in Iwate and Miyagi Prefectures or selected places arranged by the municipality and ToMMo in case of limitations with the municipal-specific health check-up venue. Urinary sodium and potassium levels were measured using an ion-selective electrode method. The spot urinary level of sodium (mEq/L) was divided by that of potassium (mEq/L) to obtain the UNa/K ratio (mEq/mEq). The participants were categorized into two groups based on the UNa/K ratio, which had a cut-off point at the 75th percentile for each sex in the study group. Therefore, a high UNa/K ratio was defined as a value at the top 75th percentile, which was 5.09 for men and 4.76 for women.

### Other measurements

The body mass index (BMI) was calculated as the weight divided by the square of the height (kg/m^2^). General medical examination data included the blood pressure levels (mmHg), estimated glomerular filtration rate (eGFR) (mL/kg/1.73 m^2^), plasma HbA1c levels (%), and serum low-density lipoprotein (LDL) cholesterol levels (mg/dL). The eGFR was calculated based on the serum creatinine level, according to the Chronic Kidney Disease Epidemiology Collaboration equation for Japanese patients [28].

Demographic, lifestyle, and disaster-related factors were assessed through a self-administered questionnaire that included information on sex, age, treatment of hypertension, smoking status, alcohol drinking status, regular exercise, psychological distress, number of cohabitants, and change in income. Smoking status was classified as current smoking or none. Alcohol drinking status was classified into two categories: drinking 3 days a week and drinking >3 days a week (defined as current drinking). Regular exercise was defined as participation in sports or other forms of physical activity at least 3 times a week and at least 30 min at a time during a participant’s leisure time. Forms of physical activity included slow walking, brisk walking, golfing, Japanese croquet, gardening, playing tennis, jogging, aerobics, and swimming. Psychological distress was evaluated using the Japanese version of the 6-item Kessler Psychological Scale (K6) [29, 30]. Based on the number of cohabitants, participants were classified as living alone or not. Regarding the change in income, participants were classified as those who had a decrease in income or those who did not. Their housing condition at the time of health checkup was classified as temporary housing (including “Living in a shelter” and “Living in temporary housing,” which were provided as answers in the questionnaire), or not.

### Statistical analyses

The one-way analysis of variance for continuous variables and the chi-square test for categorical variables were used to compare the characteristics of participants among the four groups according to the extent of house collapse. Dunnett’s test was performed for multiple comparisons using the ND group as the reference group. The value of the UNa/K ratio was log-transformed, as it had a log-normal distribution. The geometric means and 95% confidence intervals (CIs) of the UNa/K ratio were calculated for each group of house collapse using analysis of covariance and adjusted for the following factors: age, sex, BMI, current smoking, current drinking, regular exercise, living alone or not, and having a decreased income or not (Model 1); the aforementioned factors plus housing condition (Model 2); and the variables included in Model 1 plus systolic blood pressure (SBP), eGFR, HbA1c and LDL cholesterol levels, and K6 score (Model 3). We performed Pearson’s and Spearman’s correlation analysis, and confirmed that there was no collinearity between variables.

Multiple comparisons adjusted using Bonferroni correction were performed. In addition, sex- and age-adjusted and multivariableadjusted odds ratios (ORs) and 95% CIs of high UNa/K ratio in the TC, HC, PC, and ND groups were calculated using logistic regression. The same analysis was performed after excluding participants who were receiving antihypertensive treatment.

To investigate the effect of the GEJE on the UNa/K ratio over time, we divided the participants into the following three groups according to the period of the check-up: April 1, 2013, to March 31, 2014; April 1, 2014, to March 31, 2015; and April 1, 2015, to March 31, 2016. We also investigated the multivariate-adjusted geometric means of the UNa/K ratio and ORs for high UNa/K ratios in the following age groups: 20–49, 50–64, and 65–74 years. Data were analyzed using IBM SPSS Statistics Version 24.0 (IBM, Armonk, NY, USA). Two-sided *P*-values < 0.05 were considered statistically significant.

## Results

### Participant characteristics

The participants’ ages ranged from 20 to 74 years (mean age, 60.5 ± 11.14 years), and 63.3% were women. Table 1 shows the characteristics of participants according to the extent of house collapse. Of the 29 542 participants, 5 359 (18.1%), 3 576 (12.1%), 7 331 (24.8%), and 13 276 (44.9%) were in the TC, HC, PC, and ND groups, respectively. Participants whose houses collapsed during the GEJE were more likely to have high BMI and LDL cholesterol levels and low eGFR and HbA1c levels than those whose houses did not sustain any damage. Among the groups, the TC group had the highest percentage of participants with hypertension. There were no significant differences in sex and sBP levels among the four groups. Regarding lifestyle factors, participants in the TC group were more likely to be current smokers and less likely to be current alcohol drinkers and to perform regular exercise. In terms of disaster-related factors, participants whose houses collapsed were more likely to have high K6 scores and to experience a decrease in income. Among the groups, the TC group had the highest percentage of participants who were living alone or whose income decreased. The association between house collapse and the housing condition at the time of health checkup was also investigated (Supplementary Table 1). We also investigated the characteristics according to the survey year, as shown in Supplementary Table 2.

**Table 1:** Characteristics of total participants according to the extent of house collapse (*n* = 29,542)

	Extent of house collapse			
	TC	HC	PC	ND
Number of subjects	5359	3576	7331	13,276
Age, years	60.1 ± 11.3	60.6 ± 10.9	60.8 ± 10.8*	60.3 ± 11.3
Female sex, %	63.5	63.4	62.7	63.8
High UNa/K ratio, %	25.2*	27.1*	26.8*	23.5
UNa/K ratio	3.35*	3.37*	3.32*	3.23
BMI, kg/m^2^	23.7 ± 3.7*	23.6 ± 3.6*	23.4 ± 3.5	23.4 ± 3.6
Systolic blood pressure, mmHg	125.9 ± 16.9	126.4 ± 17.0	126.1 ± 17.3	126.3 ± 17.2
eGFR, mL/kg/1.73 m^2^	80.2 ± 16.9	77.5 ± 15.4*	76.9 ± 15.4*	80.3 ± 17.1
HbA1c, %	5.66 ± 0.58*	5.61 ± 0.58*	5.62 ± 0.59*	5.68 ± 0.62
LDL cholesterol, mg/dL	123.4 ± 31.3*	122.6 ± 31.4*	124.0 ± 31.6*	120.5 ± 30.7
Current smoker, %	15.8*	14.1	13.3	13.0
Current drinker (>3 days per week), %	28.6*	32.2	31.7	30.5
Doing regular exercise (>3 × 30 min per week), %	38.4*	42.3	43.3	41.6
K6 score	5.6 ± 4.9*	4.8 ± 4.4*	4.6 ± 4.4*	4.3 ± 4.4
Living alone, %	10.8*	7.5	6.6*	8.8
Decrease of income, %	27.4*	20.0*	17.0*	14.6
Treatment of hypertension, %	29.3*	27.4	26.0	27.0

Values except for the UNa/K ratio are presented as means ± standard deviation or as percentages. The UNa/K ratio is presented as the geometric mean, because of its log-normal distribution. Multiple comparisons were performed using Dunnett’s test for continuous variables and Fisher’s test adjusted by Bonferroni correction for categorical variables

*TC* total collapse, *HC* half collapse, *PC* partial collapse, *ND* no damage, *UNa/K ratio* urine sodium-to-potassium ratio, *BMI* body mass index, *eGFR* estimated glomerular filtration rate, *HbA1c* hemoglobin A1c, *LDL* low-density lipoprotein

*P  <  0.05 versus ND

### Association between UNa/K and the extent of house collapse

Table 2 shows the multivariate-adjusted geometric means of the UNa/K ratio according to the extent of house collapse. In the total study population, the multivariate-adjusted geometric means (95% CIs) of the UNa/K ratios for participants whose houses underwent TC, HC, and PC were 3.33 (3.28–3.38), 3.37 (3.30–3.43), and 3.32 (3.28–3.37), respectively, according to Model 1, all of which were significantly greater than that of those whose houses had ND (3.24 [3.21–3.27]). The same tendency was observed for both sexes. The values obtained based on Models 2 or 3 were very similar to those of Model 1; therefore, we refer to the values of Model 1 alone from here onward.

**Table 2:** Multivariate adjusted geometric means (95% confidence intervals) of the UNa/K ratio according to the extent of house collapse

	Extent of house collapse			
	TC	HC	PC	ND
Total (*n* = 29,542)				
Number of participants	5359	3576	7331	13,276
Sex- and age-adjusted geometric mean (95% CI)	3.35 (3.29–3.40)	3.37 (3.31–3.44)	3.32 (3.28–3.36)	3.23 (3.20–3.27)
*P*-value	0.002	<0.001	0.010	
Multivariate-adjusted geometric mean (Model 1) (95% CI)	3.33 (3.28–3.38)	3.37 (3.30–3.43)	3.32 (3.28–3.37)	3.24 (3.21–3.27)
*P*-value	0.020	0.003	0.018	
Multivariate-adjusted geometric mean (Model 2) (95% CI)	3.34 (3.28–3.41)	3.37 (3.30–3.43)	3.32 (3.28–3.37)	3.24 (3.21–3.27)
*P*-value	0.049	0.002	0.018	
Multivariate-adjusted geometric mean (Model 3) (95% CI)	3.32 (3.27–3.38)	3.38 (3.32–3.44)	3.35 (3.31–3.39)	3.23 (3.19–3.26)
*P*-value	0.007	<0.001	<0.001	
Men (*n* = 10,805)				
Number of participants	1958	1309	2738	4800
Age-adjusted geometric mean (95% CI)	3.49 (3.39–3.56)	3.49 (3.39–3.60)	3.50 (3.42–3.56)	3.35 (3.29–3.42)
*P*-value	0.094	0.135	0.011	
Multivariate-adjusted geometric mean (Model 1) (95% CI)	3.48 (3.39–3.57)	3.49 (3.38–3.60)	3.50 (3.43–3.58)	3.35 (3.29–3.40)
*P*-value	0.114	0.167	0.007	
Multivariate-adjusted geometric mean (Model 2) (95% CI)	3.48 (3.37–3.60)	3.48 (3.38–3.60)	3.50 (3.43–3.58)	3.35 (3.29–3.40)
*P*-value	0.263	0.164	0.007	
Multivariate-adjusted geometric mean (Model 3) (95% CI)	3.46 (3.38–3.55)	3.50 (3.39–3.61)	3.53 (3.46–3.61)	3.34 (3.28–3.39)
*P*-value	0.097	0.0502	<0.001	
Women (*n* = 18,737)				
Number of participants	3401	2267	4593	8476
Age-adjusted geometric mean (95% CI)	3.27 (3.19–3.35)	3.31 (3.22–3.39)	3.22 (3.16–3.29)	3.17 (3.13–3.22)
*P*-value	0.036	0.009	0.747	
Multivariate-adjusted geometric mean (Model 1) (95% CI)	3.25 (3.19–3.31)	3.30 (3.23–3.38)	3.22 (3.17–3.28)	3.18 (3.14–3.22)
*P*-value	0.329	0.025	1.000	
Multivariate-adjusted geometric mean (Model 2) (95% CI)	3.26 (3.18–3.34)	3.30 (3.23–3.38)	3.22 (3.17–3.28)	3.17 (3.14–3.22)
*P*-value	0.422	0.024	1.000	
Multivariate-adjusted geometric mean (Model 3) (95% CI)	3.25 (3.19–3.31)	3.31 (3.24–3.39)	3.25 (3.19–3.30)	3.16 (3.13–3.20)
*P*-value	0.156	0.003	0.075	

Multivariate-adjusted geometric means (95% CIs) (Model 1) of the UNa/K ratio were calculated by analysis of covariance with adjustment for age, sex, body mass index, current smoking, current drinking and regular exercise, living alone or not, and having a decreased income or not

Multivariate-adjusted geometric means (95% CIs) (Model 2) of the UNa/K ratio were calculated by analysis of covariance with adjustment for age, sex, body mass index, current smoking, current drinking and regular exercise, living alone or not, having a decreased income or not, and residential state

Multivariate-adjusted geometric means (95% CIs) (Model 3) of the UNa/K ratio were calculated by analysis of covariance with adjustment for age, sex, body mass index, systolic blood pressure, estimated glomerular filtration rate, hemoglobin A1c and low-density lipoprotein cholesterol levels, K6 score, current smoking, current drinking and regular exercise, living alone or not, and having a decreased income or not

Multiple comparisons adjusted by Bonferroni correction were performed

*UNa/K ratio* urine sodium-to-potassium ratio, *TC* total collapse, *HC* half collapse, *PC* partial collapse, *ND* no damage, *CI* confidence interval

Table 3 shows the ORs and 95% CIs for high UNa/K ratios using logistic regression. For all participants, the extent of house collapse was associated with a high UNa/K ratio, especially in the PC and HC groups, with multivariable-adjusted ORs of 1.20 (95% CI, 1.12–1.28) and 1.20 (95% CI, 1.11–1.31), respectively. The same tendency as that of the overall group was observed for both sexes. The subgroup without antihypertensive treatment also showed the same tendency as that of the overall group (Supplementary Tables 3 and 4).

**Table 3:** Multivariate-adjusted odds ratios (95% confidence intervals) for high UNa/K ratios in participants according to the extent of house collapse

	Extent of house collapse			
	TC	HC	PC	ND
Total (*n* = 29,542)				
Number of participants	5359	3576	7331	13,276
Number of cases with a high UNa/K ratio	1350	970	1964	3124
Crude OR (95% CI)	1.09 (1.02–1.18)	1.21 (1.11–1.32)	1.19 (1.11–1.27)	Reference
*P*-value	0.016	<0.001	<0.001	
Sex- and age-adjusted OR (95% CI)	1.09 (1.02–1.18)	1.21 (1.12–1.32)	1.19 (1.12–1.28)	Reference
*P*-value	0.019	<0.001	<0.001	
Multivariate-adjusted OR (95% CI) (Model 1)	1.07 (0.99–1.15)	1.20 (1.11–1.31)	1.20 (1.12–1.28)	Reference
*P*-value	0.074	<0.001	<0.001	
Multivariate-adjusted OR (95% CI) (Model 2)	1.08 (0.99–1.19)	1.20 (1.11–1.31)	1.20 (1.12–1.28)	Reference
*P*-value	0.087	<0.001	<0.001	
Multivariate-adjusted OR (95% CI) (Model 3)	1.08 (0.997–1.16)	1.22 (1.12–1.33)	1.23 (1.15–1.31)	Reference
*P*-value	0.058	<0.001	<0.001	
Men (*n* = 10,805)				
Number of participants	1958	1309	2738	4800
Number of cases with a high UNa/K ratio	491	363	731	1121
Crude OR (95% CI)	1.10 (0.97–1.24)	1.26 (1.10–1.45)	1.20 (1.07–1.33)	Reference
*P*-value	0.132	0.001	0.001	
Age-adjusted OR (95% CI)	1.09 (0.97–1.23)	1.26 (1.10–1.45)	1.20 (1.08–1.34)	Reference
*P*-value	0.167	0.001	<0.001	
Multivariate-adjusted OR (95% CI) (Model 1)	1.08 (0.96–1.23)	1.26 (1.10–1.45)	1.22 (1.09–1.36)	Reference
*P*-value	0.211	0.001	<0.001	
Multivariate-adjusted OR (95% CI) (Model 2)	1.06 (0.91–1.24)	1.26 (1.09–1.44)	1.22 (1.09–1.36)	Reference
*P*-value	0.430	0.001	<0.001	
Multivariate-adjusted OR (95% CI) (Model 3)	1.08 (0.95–1.22)	1.27 (1.11–1.46)	1.25 (1.12–1.39)	Reference
*P*-value	0.233	<0.001	<0.001	
Women (*n* = 18,737)				
Number of participants	2401	2267	4593	8476
Number of cases with a high UNa/K ratio	859	607	1233	2003
Crude OR (95% CI)	1.09 (0.996–1.20)	1.18 (1.06–1.31)	1.19 (1.09–1.29)	Reference
*P*-value	0.061	0.002	<0.001	
Age-adjusted OR (95% CI)	1.09 (0.998–1.20)	1.19 (1.07–1.32)	1.19 (1.10–1.29)	Reference
*P*-value	0.056	0.002	<0.001	
Multivariate-adjusted OR (95% CI) (Model 1)	1.06 (0.97–1.17)	1.18 (1.06–1.31)	1.19 (1.09–1.29)	Reference
*P*-value	0.200	0.003	<0.001	
Multivariate-adjusted OR (95% CI) (Model 2)	1.10 (0.98–1.23)	1.18 (1.06–1.31)	1.19 (1.09–1.29)	Reference
*P*-value	0.121	0.002	<0.001	
Multivariate-adjusted OR (95% CI) (Model 3)	1.07 (0.98–1.18)	1.20 (1.08–1.33)	1.22 (1.12–1.33)	Reference
*P*-value	0.150	0.001	<0.001	

Multivariate-adjusted ORs (Model 1) for high UNa/K ratios were calculated using a logistic regression model with adjustment for age, sex, body mass index, current smoking, current drinking and regular exercise, living alone or not, and having a decreased income or not

Multivariate-adjusted ORs (Model 2) for high UNa/K ratios were calculated using a logistic regression model with adjustment for age, sex, body mass index, current smoking, current drinking and regular exercise, living alone or not, having a decreased income or not, and residential state

Multivariate-adjusted ORs (Model 3) for high UNa/K ratios were calculated using a logistic regression model with adjustment for age, sex, body mass index, systolic blood pressure, estimated glomerular filtration rate, hemoglobin A1c and low-density lipoprotein cholesterol levels, K6 score, current smoking, current drinking and regular exercise, living alone or not, and having a decreased income or not

The cutoff was the 75th percentile in UNa/K ratio for each sex in the study group. The UNa/K ratio in the 75th percentile or higher was defined as “high UNa/K ratio.” The 75th percentile in UNa/K ratio was 5.09% for men and 4.76% for women

*UNa/K ratio* urine sodium-to-potassium ratio, *TC* total collapse, *HC* half collapse, *PC* partial collapse, *ND* no damage, *OR* odds ratio, *CI* confidence interval

### Effect of the GEJE on the UNa/K ratio over time

Supplementary Tables 5 and 6 show the multivariate-adjusted geometric means of the UNa/K ratio and ORs for high UNa/K ratios according to the period of surveys, respectively. A similar tendency as in the entire survey period was observed in the first and second years of the survey (i.e., fiscal years 2013 and 2014), although it was not noted in the third year (i.e., fiscal year 2015).

### Stratified analysis by age group

Supplementary Tables 7 and 8 show the respective multivariate-adjusted geometric means of the UNa/K ratio and ORs for high UNa/K ratios in the following age groups: 20–49, 50–64, and 65–74 years. Among the age groups, the same tendency as that in the overall study participants was observed in the groups aged 50–64 and 65–74 years but not in the group aged 20–49 years.

## Discussion

Based on the analyses of cross-sectional data on the victims of the 2011 GEJE who were living in the coastal areas, the multivariate-adjusted geometric means of the UNa/K ratio or ORs for high UNa/K ratios were significantly higher in victims who experienced house collapse than in those who did not. The same tendency was observed for both sexes after excluding those who were receiving antihypertensive treatment. Based on the results of the stratified analysis according to the investigation period, the same tendency as that in the entire period was observed in the first and second years of investigation (fiscal years 2013 and 2014), although it was not observed in the third year (fiscal year 2015). This finding suggests that the effect of the disaster on the UNa/K ratio might have remained stronger in the immediate period following the GEJE.

Several reports have indicated an imbalance in nutritional intake after an earthquake [24, 31, 32]. People living in difficult conditions were likely to have a lower prudent dietary pattern score within 1 year after the 2011 GEJE [24]. In this study, we concluded that nutritional intake was more important than the housing condition because the results of the multivariable-adjusted analysis changed negligibly on adding housing condition as an adjustment variable, indicating that the housing condition did not have much effect on UNa/K. The limited supply of fresh fruits and vegetables to affected areas might also be the cause of vitamin C deficiency observed within 4 weeks in previously healthy people [32]. However, these studies were unable to elucidate the effect of a natural disaster on sodium or potassium intake. To the best of our knowledge, this study is the first to demonstrate the association between a natural disaster and sodium and potassium intake in a large cohort of community dwellers.

Because this study was cross-sectional in nature, we could not conclude causal relationships. However, we can infer several possible mechanisms for our results. First, experiencing a house collapse could change a victim’s living environment, forcing them to consume foods that are high in sodium and low in potassium. One month after the GEJE, the victims might have encountered difficulties in obtaining and preparing meals due to shortages of cooking equipment, utilities, and food [23]. Previous studies have shown that victims living in shelters or temporary housing tend to be immobile [33], to gain weight [33, 34], and to change their dietary patterns [35]. Second, lower socioeconomic status (SES) has been reported to be associated with a higher UNa/K ratio because lower SES might be associated with less consumption of fruits and vegetables [36]. In this study, a large proportion of participants whose houses collapsed during the GEJE underwent a decrease in income. The house collapse event might have negatively affected the victims’ SES, including their income, and this may partly account for the association between house collapse and UNa/K ratio. Relocation after a house collapse might have also deteriorated the victims’ SES. In a study involving 6 528 survivors of the GEJE, relocation was associated with several disaster-related unhealthy conditions and lifestyles, including smoking, physical inactivity, psychological distress, and socioeconomic deprivation [37]. Third, psychological distress is also one of the factors known to be associated with poor dietary intake; for example, people with depression tend not to consume fresh foods [38, 39]. In our study, participants who experienced house collapse showed significantly higher K6 scores, indicating worse mental health status. Therefore, mental health problems might have also aggravated the dietary and nutritional intake of participants who experienced house collapse during the GEJE.

This study had some limitations. First, the study used spot urine samples instead of 24-h urine samples; thus, the electrolyte levels may fluctuate depending on the collection time, which might lead to underestimated values. However, several recent epidemiological studies [21, 40] used spot urine samples instead of 24-h urine samples to estimate usual sodium or potassium intake. These studies succeeded in elucidating the traditional correlations between the UNa/K ratio in spot urine and CVD risk or hypertension [21, 40]. Therefore, the use of spot urine samples to determine the UNa/K ratio is appropriate for population analysis. Second, the participants in this study comprised those who underwent health check-ups. This could mean that they might have been more conscious of their health than the general population living in the same area. Therefore, the mean levels of the UNa/K ratio might have been underestimated compared with those of the general population living in the same area. Third, the living status might have also affected the UNa/K ratio. The participants in temporary housing may have been receiving full support regarding their physical and mental health or daily living needs, which may partly account for the underestimation of the TC group. Fourth, as an indicator for SES, we focused on the decrease in income but not on the absolute income level, because the questionnaire did not include this parameter as an item. Lastly, as mentioned above, this study was a cross-sectional study; thus, a causal relationship cannot be inferred.

In summary, this study revealed that victims whose houses collapsed due to the 2011 GEJE had a significantly higher UNa/K ratio than those whose houses did not sustain damage. Our findings suggest that victims who experienced a natural disaster tend to have an unbalanced diet in terms of the sodium and potassium ratio. Therefore, disaster victims should receive nutritional interventions comprising a diet with low sodium-to-potassium ratio, including fresh fruits and vegetables, to maintain a healthy status and to prevent hypertension or CVD. Future studies using controlled trials should confirm whether nutritional interventions benefit disaster victims by preventing CVD incidence.

## Supplementary information

Supplemental Material

## Data Availability

All data relevant to the study are included in the article or uploaded as supplementary information.

## References

[1] Trichopoulos D, Katsouyanni K, Zavitsanos X, Tzonou A, Dalla-Vorgia P (1983). Psychological stress and fatal heart attack: the Athens (1981) earthquake natural experiment. Lancet.

[2] Katsouyanni K, Kogevinas M, Trichopoulos D (1986). Earthquake-related stress and cardiac mortality. Int J Epidemiol.

[3] Ogawa K, Tsuji I, Shiono K, Hisamichi S (2000). Increased acute myocardial infarction mortality following the 1995 Great Hanshin-Awaji earthquake in Japan. Int J Epidemiol.

[4] Kloner RA, Leor J, Poole WK, Perritt R (1997). Population-based analysis of the effect of the Northridge Earthquake on cardiac death in Los Angeles County, California. J Am Coll Cardiol.

[5] Watanabe H, Kodama M, Okura Y, Aizawa Y, Tanabe N, Chinushi M (2005). Impact of earthquakes on Takotsubo cardiomyopathy. JAMA.

[6] Leor J, Poole WK, Kloner RA (1996). Sudden cardiac death triggered by an earthquake. N. Engl J Med.

[7] Becquart NA, Naumova EN, Singh G, Chui KKH (2018). Cardiovascular disease hospitalizations in Louisiana parishes’ elderly before, during and after Hurricane Katrina. Int J Environ Res Public Health.

[8] Peters MN, Moscona JC, Katz MJ, Deandrade KB, Quevedo HC, Tiwari S (2014). Natural disasters and myocardial infarction: the six years after Hurricane Katrina. Mayo Clin Proc.

[9] Nakagawa I, Nakamura K, Oyama M, Yamazaki O, Ishigami K, Tsuchiya Y (2009). Long-term effects of the Niigata-Chuetsu earthquake in Japan on acute myocardial infarction mortality: an analysis of death certificate data. Heart.

[10] Omama S, Yoshida Y, Ogasawara K, Ogawa A, Ishibashi Y, Nakamura M (2013). Influence of the great East Japan earthquake and tsunami 2011 on occurrence of cerebrovascular diseases in Iwate, Japan. Stroke.

[11] Yamauchi H, Yoshihisa A, Iwaya S, Owada T, Sato T, Suzuki S (2013). Clinical features of patients with decompensated heart failure after the Great East Japan Earthquake. Am J Cardiol.

[12] Nakamura M, Tanaka F, Nakajima S, Honma M, Sakai T, Kawakami M (2012). Comparison of the incidence of acute decompensated heart failure before and after the major tsunami in Northeast Japan. Am J Cardiol.

[13] Tanaka F, Makita S, Ito T, Onoda T, Sakata K, Nakamura M (2015). Relationship between the seismic scale of the 2011 northeast Japan earthquake and the incidence of acute myocardial infarction: a population-based study. Am Heart J.

[14] Omama S, Yoshida Y, Ogasawara K, Ogawa A, Ishibashi Y, Nakamura M (2014). Extent of flood damage increased cerebrovascular disease incidences in Iwate Prefecture after the great East Japan earthquake and tsunami of 2011. Cerebrovasc Dis.

[15] Nakamura M, Tanaka K, Tanaka F, Matsuura Y, Komi R, Niiyama M (2017). Long-term effects of the 2011 Japan earthquake and tsunami on incidence of fatal and nonfatal myocardial infarction. Am J Cardiol.

[16] Nakamura M, Tanaka F, Komi R, Tanaka K, Onodera M, Kawakami M (2016). Sustained increase in the incidence of acute decompensated heart failure after the 2011 Japan earthquake and tsunami. Am J Cardiol.

[17] Kawasaki T, Delea CS, Bartter FC, Smith H (1978). The effect of high-sodium and low-sodium intakes on blood pressure and other related variables in human subjects with idiopathic hypertension. Am J Med.

[18] Adrogué HJ, Madias NE (2014). The impact of sodium and potassium on hypertension risk. Semin Nephrol.

[19] Thi Minh Nguyen T, Miura K, Tanaka-Mizuno S, Tanaka T, Nakamura Y, Fujiyoshi A (2019). Association of blood pressure with estimates of 24-h urinary sodium and potassium excretion from repeated single-spot urine samples. Hypertens Res.

[20] Liu HH, Gao XM, Li Y, Wu Y, Zhou L, Mai JZ (2018). Relationship between overnight urinary sodium to potassium ratio and the risk of cardiovascular disease. Zhonghua Xin Xue Guan Bing Za Zhi.

[21] Tabara Y, Takahashi Y, Kumagai K, Setoh K, Kawaguchi T, Takahashi M (2015). Descriptive epidemiology of spot urine sodium-to-potassium ratio clarified close relationship with blood pressure level: the Nagahama study. J Hypertens.

[22] Cook NR, Obarzanek E, Cutler JA, Buring JE, Rexrode KM, Kumanyika SK (2009). Joint effects of sodium and potassium intake on subsequent cardiovascular disease: the trials of hypertension prevention follow-up study. Arch Intern Med.

[23] Tsuboyama-Kasaoka N, Hoshi Y, Onodera K, Mizuno S, Sako K (2014). What factors were important for dietary improvement in emergency shelters after the Great East Japan Earthquake?. Asia Pac J Clin Nutr.

[24] Nishi N, Yoshimura E, Ishikawa-Takata K, Tsuboyama-Kasaoka N, Kubota T, Miyachi M (2013). Relationship of living conditions with dietary patterns among survivors of the great East Japan earthquake. J Epidemiol.

[25] von Elm E, Altman DG, Egger M, Pocock SJ, Gøtzsche PC, Vandenbroucke JP (2014). The Strengthening the Reporting of Observational Studies in Epidemiology (STROBE) Statement: guidelines for reporting observational studies. Int J Surg.

[26] Hozawa A, Tanno K, Nakaya N, Nakamura T, Tsuchiya N, Hirata T (2021). Study profile of the Tohoku Medical Megabank Community-Based Cohort Study. J Epidemiol.

[27] Kuriyama S, Yaegashi N, Nagami F, Arai T, Kawaguchi Y, Osumi N (2016). The Tohoku Medical Megabank Project: design and mission. J Epidemiol.

[28] Matsuo S, Imai E, Horio M, Yasuda Y, Tomita K, Nitta K (2009). Revised equations for estimated GFR from serum creatinine in Japan. Am J Kidney Dis.

[29] Kessler RC, Barker PR, Colpe LJ, Epstein JF, Gfroerer JC, Hiripi E (2003). Screening for serious mental illness in the general population. Arch Gen Psychiatry.

[30] Furukawa TA, Kawakami N, Saitoh M, Ono Y, Nakane Y, Nakamura Y (2008). The performance of the Japanese version of the K6 and K10 in the World Mental Health Survey Japan. Int J Methods Psychiatr Res.

[31] Nozue M, Nishi N, Tsubota-Utsugi M, Miyoshi M, Yonekura Y, Sakata K (2017). Combined associations of physical activity and dietary intake with health status among survivors of the Great East Japan Earthquake. Asia Pac J Clin Nutr.

[32] Amagai T, Ichimaru S, Tai M, Ejiri Y, Muto A (2014). Nutrition in the great East Japan earthquake disaster. Nutr Clin Pr.

[33] Takahashi S, Ishiki M, Kondo N, Ishiki A, Toriyama T, Takahashi S (2015). Health effects of a farming program to foster community social capital of a temporary housing complex of the 2011 great East Japan earthquake. Disaster Med Public Health Prep.

[34] Takahashi S, Yonekura Y, Sasaki R, Yokoyama Y, Tanno K, Sakata K (2016). Weight gain in survivors living in temporary housing in the tsunami-stricken area during the recovery phase following the Great East Japan Earthquake and Tsunami. PLoS One.

[35] Ishii T, Ochi S, Tsubokura M, Kato S, Tetsuda T, Kato J (2015). Physical performance deterioration of temporary housing residents after the Great East Japan Earthquake. Prev Med Rep..

[36] Miyagawa N, Okuda N, Nakagawa H, Takezaki T, Nishi N, Takashima N (2018). Socioeconomic status associated with urinary sodium and potassium excretion in Japan: NIPPON DATA2010. J Epidemiol.

[37] Takahashi S, Nakamura M, Yonekura Y, Tanno K, Sakata K, Ogawa A (2016). Association between relocation and changes in cardiometabolic risk factors: a longitudinal study in tsunami survivors of the 2011 Great East Japan Earthquake. BMJ Open.

[38] Lai JS, Hiles S, Bisquera A, Hure AJ, McEvoy M, Attia J (2014). A systematic review and meta-analysis of dietary patterns and depression in community-dwelling adults. Am J Clin Nutr.

[39] Sanhueza C, Ryan L, Foxcroft DR (2013). Diet and the risk of unipolar depression in adults: systematic review of cohort studies. J Hum Nutr Diet.

[40] Iwahori T, Miura K, Ueshima H, Tanaka-Mizuno S, Chan Q, Arima H (2019). Urinary sodium-to-potassium ratio and intake of sodium and potassium among men and women from multiethnic general populations: the INTERSALT Study. Hypertens Res.

