# Supplementary material for: Association between the extent of house collapse and urine sodium-to-potassium ratio of victims affected by the 2011 Great East Japan Earthquake and Tsunami: a cross-sectional study
Source: Hypertens Res. 2023 Feb 17;46(5):1247–56. doi: 10.1038/s41440-023-01190-5 (PMC10164643; doi:10.1038/s41440-023-01190-5)
Supplement: Supplementary file 1 — Supplemental Material [file 41440_2023_1190_MOESM1_ESM.docx]

**Supplemental Material**

**Supplementary Table 1. Number of participants and percentage of each** **residential state according to the extent of house collapse**

|  | **Extent of house collapse** | | | | **Total** |
| --- | --- | --- | --- | --- | --- |
|  | **TC** | **HC** | **PC** | **ND** |  |
| Living in a shelter | 2 177 (40.6%) | 98 (2.7%) | 8 (0.1%) | 22 (0.2%) | 2 305 (7.8%) |
| Living in a temporary housing | 508 (9.5%) | 63 (1.8%) | 17 (0.2%) | 87 (0.7%) | 675 (2.3%) |
| Moved to rental housing | 109 (2.0%) | 52 (1.5%) | 51 (0.7%) | 146 (1.1%) | 358 (1.2%9 |
| Living in an acquaintance’s house | 133 (2.5%) | 47 (1.3%) | 65 (0.9%) | 190 (1.4%) | 435 (1.5%) |
| Rebuilt a new house where they had been living | 498 (9.3%) | 427 (11.9%) | 76 (1.0%) | 14 (0.1%) | 1 015 (3.4%) |
| Rebuilt a new house in a different place than where they had been living | 1079 (20.1%) | 96 (2.7%) | 60 (0.8%) | 166 (1.3%) | 1 401 (4.7%) |
| Living in the same house as before the GEJE | 400 (7.5%) | 2 637 (73.7%) | 6 869 (93.7%) | 11 968 (90.1%) | 21 874 (74.0%) |
| Others | 455 (8.5%) | 156 (4.4%) | 185 (2.5%) | 683 (5.1%) | 1 479 (5.0%) |
| Total | 5 359 (100.0%) | 3 576 (100.0%) | 7 331 (100.0%) | 13 276 (100.0%) | 29 542 (100.0%) |

The residential state at the time of health checkup according to the extent of house collapse were investigated. Especially, 50.1% of those in the TC group lived in shelters or temporary housing at the timepoint of the survey, whereas the corresponding percentage in the HC, PC, and ND groups was <5%. The percentage of participants living in the same house where they had been living before the GEJE was only 7.5% in the TC group, whereas that in the HC, PC, and ND groups was >70%.

TC, total collapse; HC, half collapse; PC, partial collapse; ND, no damage; CI, confidence interval; GEJE, Great East Japan Earthquake

**Supplementary Table 2.** **Characteristics of the participants according to the year of study enrollment**

|  | **Fiscal year of the survey** | | |
| --- | --- | --- | --- |
|  | **2013** | **2014** | **2015** |
| Number of participants | 9 998 | 13 900 | 5644 |
| Age, years | 60.3 (10.8) | 61.2 (10.8) * | 60.5 (11.1) * |
| Female sex, % | 63.1 | 63.4 | 64.1 |
| High UNa/K ratio, % | 27.3 | 25.3* | 20.5* |
| UNa/K ratio | 3.44 (3.40–3.48) | 3.28 (3.25–3.31) * | 3.08 (3.03–3.12) * |
| BMI, kg/m^2^ | 23.6 (3.5) | 23.4 (3.6) * | 23.4 (3.67) * |
| Systolic blood pressure, mmHg | 127.8 (17.0) | 125.5 (16.8) * | 124.9 (17.1) * |
| eGFR, mL/kg/1.73 m^2^ | 78.1 (15.7) | 79.4 (17.0) * | 80.4 (16.7) * |
| HbA1c, % | 5.59 (0.63) | 5.68 (0.59) * | 5.70 (0.55) * |
| LDL cholesterol, mg/dL | 120.7 (31.4) | 123.0 (31.2) * | 122.5 (30.5) * |
| Current smoker, % | 14.4 | 12.7* | 15.0 |
| Current drinker (>3 days per week), % | 30.0 | 31.3 | 30.2 |
| Doing regular exercise (>3 × 30 minutes per week), % | 39.8 | 43.9* | 38.8 |
| K6 score | 4.7 (4.6) | 4.6 (4.4) | 4.7 (4.5) |
| Living alone, % | 9.2 | 9.1* | 9.2 |
| Decrease in income, % | 17.7 | 17.4 | 20.8* |
| Treatment of hypertension, % | 27.2 | 27.8 | 26.1 |

The characteristics of the participants according to the year of study enrollment are shown in the table. Values except for the UNa/K ratio are presented as means ± standard deviations or as percentages. The UNa/K ratio is presented as medians (interquartile ranges). Multiple comparisons were performed using Dunnett’s test for continuous variables and Fisher’s test adjusted using Bonferroni correction for categorical variables.

**P*<0.05 versus 2013.

UNa/K ratio, urine sodium-to-potassium ratio; BMI, body mass index; eGFR, estimated glomerular filtration rate; HbA1c, hemoglobin A1c; LDL, low-density lipoprotein; K6, 6-item Kessler Psychological Scale

**Supplemental Table 3**. Multivariate adjusted geometric means (95% confidence intervals) of the UNa/K ratio according to the extent of house collapse among participants without antihypertensive treatment

|  | **Extent of house collapse** | | | |
| --- | --- | --- | --- | --- |
|  | **TC** | **HC** | **PC** | **ND** |
| Total (n=21 495) |  |  |  |  |
| Number of participants | 3 787 | 2 598 | 5 423 | 9 687 |
| Sex- and age-adjusted geometric mean (95% CI) | 3.35 (3.28–3.40) | 3.35 (3.27–3.42) | 3.30 (3.25–3.35) | 3.23 (3.20–3.27) |
| *P*-value | 0.008 | 0.036 | 0.240 |  |
| Multivariate-adjusted geometric mean (95% CI) | 3.32 (3.26–3.38) | 3.35 (3.28–3.42) | 3.32 (3.28–3.37) | 3.23 (3.19–3.27) |
| *P*-value | 0.087 | 0.015 | 0.013 |  |
|  |  |  |  |  |
| Men (n=7 125) |  |  |  |  |
| Number of participants | 1 256 | 860 | 1 834 | 3 175 |
| Age-adjusted geometric mean (95% CI) | 3.45 (3.34–3.55) | 3.48 (3.36–3.62) | 3.48 (3.39–3.57) | 3.32 (3.25–3.38) |
| *P*-value | 0.261 | 0.135 | 0.025 |  |
| Multivariate-adjusted geometric mean (95% CI) | 3.43 (3.33–3.54) | 3.49 (3.36–3.62) | 3.50 (3.41–3.59) | 3.31 (3.25–3.37) |
| *P*-value | 0.318 | 0.080 | 0.004 |  |
|  |  |  |  |  |
| Women (n=14 370) |  |  |  |  |
| Number of participants | 2 531 | 1 738 | 2 639 | 6 512 |
| Age-adjusted geometric mean (95% CI) | 3.30 (3.23–3.37) | 3.28 (3.19–3.37) | 3.21 (3.15–3.27) | 3.19 (3.15–3.24) |
| *P*-value | 0.074 | 0.475 | 1.000 |  |
| Multivariate-adjusted geometric mean (95% CI) | 3.26 (3.19–3.33) | 3.29 (3.20–3.37) | 3.24 (3.18–3.30) | 3.19 (3.15–3.23) |
| *P*-value | 0.685 | 0.304 | 1.000 |  |

Multivariate-adjusted geometric means (95% CI) of the UNa/K ratio were calculated by analysis of covariance with adjustment for age, sex, body mass index, systolic blood pressure, estimated glomerular filtration rate, hemoglobin A1c and low-density lipoprotein cholesterol levels, K6 score, current smoking, current drinking and regular exercise, living alone or not, and having a decreased income or not. Multiple comparisons adjusted by Bonferroni correction were performed.

UNa/K ratio, urine sodium-to-potassium ratio; TC, total collapse; HC, half collapse; PC, partial collapse; ND, no damage; CI, confidence interval

**Supplemental Table 4**. Multivariate-adjusted odds ratios (95% confidence intervals) for high UNa/K ratios in participants according to the extent of house collapse among participants without antihypertensive treatment

|  | **Extent of house collapse** | | | |
| --- | --- | --- | --- | --- |
|  | **TC** | **HC** | **PC** | **ND** |
| Total (n=21 495) |  |  |  |  |
| Number of participants | 3 787 | 2 598 | 5 423 | 9 687 |
| Number of cases with a high UNa/K ratio | 928 | 689 | 1. 422 | 2 213 |
| Crude OR (95% CI) | 1.10 (1.004–1.20) | 1.22 (1.10–1.35) | 1.20 (1.11–1.30) | Reference |
| *P*-value | 0.041 | <0.001 | <0.001 |  |
| Sex- and age-adjusted OR (95% CI) | 1.09 (0.996–1.19) | 1.23 (1.11–1.35) | 1.21 (1.12–1.31) | Reference |
| *P*-value | 0.053 | <0.001 | <0.001 |  |
| Multivariate-adjusted OR (95% CI) | 1.05 (0.96–1.15) | 1.23 (1.11–1.36) | 1.24 (1.14–1.34) | Reference |
| *P*-value | 0.245 | <0.001 | <0.001 |  |
|  |  |  |  |  |
| Men (n=7 125) |  |  |  |  |
| Number of participants | 1 256 | 860 | 1 834 | 3 175 |
| Number of cases with a high UNa/K ratio | 304 | 235 | 472 | 688 |
| Crude OR (95% CI) | 1.15 (0.99–1.35) | 1.36 (1.14–1.61) | 1.25 (1.10–1.43) | Reference |
| *P*-value | 0.068 | <0.001 | 0.001 |  |
| Age-adjusted OR (95% CI) | 1.14 (0.97–1.33) | 1.36 (1.14–1.62) | 1.27 (1.11–1.45) | Reference |
| *P*-value | 0.102 | <0.001 | <0.001 |  |
| Multivariate-adjusted OR (95% CI) | 1.12 (0.95–1.31) | 1.36 (1.14–1.62) | 1.29 (1.12–1.48) | Reference |
| *P*-value | 0.171 | <0.001 | <0.001 |  |
|  |  |  |  |  |
| Women (n=14 370) |  |  |  |  |
| Number of participants | 2 531 | 1 738 | 2 639 | 6 512 |
| Number of cases with a high UNa/K ratio | 624 | 454 | 950 | 1 525 |
| Crude OR (95% CI) | 1.07 (0.96–1.19) | 1.16 (1.02–1.31) | 1.18 (1.07–1.29) | Reference |
| *P*-value | 0.215 | 0.019 | <0.001 |  |
| Age-adjusted OR (95% CI) | 1.07 (0.96–1.19) | 1.16 (1.03–1.32) | 1.19 (1.08–1.30) | Reference |
| *P*-value | 0.222 | 0.014 | <0.001 |  |
| Multivariate-adjusted OR (95% CI) | 1.02 (0.92–1.14) | 1.17 (1.04–1.32) | 1.21 (1.10–1.34) | Reference |
| *P*-value | 0.662 | 0.012 | <0.001 |  |

Multivariate-adjusted odds ratios (ORs) for high UNa/K ratios were calculated using a logistic regression model with adjustment for age, sex, body mass index, systolic blood pressure, estimated glomerular filtration rate, hemoglobin A1c and low-density lipoprotein cholesterol levels, K6 score, current smoking, current drinking and regular exercise, living alone or not, and having a decreased income or not. The cutoff was the 75th percentile in UNa/K ratio for each sex in the study group. The UNa/K ratio in the 75th percentile or higher was defined as “high UNa/K ratio.” The 75th percentile in UNa/K ratio was 5.09% for men and 4.76% for women.

UNa/K ratio, urine sodium-to-potassium ratio; TC, total collapse; HC, half collapse; PC, partial collapse; ND, no damage; OR, odds ratio; CI, confidence interval

**Supplemental Table 5**. Multivariate-adjusted geometric means (95% confidence intervals) of the UNa/K ratio according to different periods of investigation

|  | **Extent of house collapse** | | | |
| --- | --- | --- | --- | --- |
|  | **TC** | **HC** | **PC** | **ND** |
| Total (n=29 542) |  |  |  |  |
| 2013 fiscal year |  |  |  |  |
| Number of participants | 1 536 | 1 281 | 2 360 | 4 821 |
| Multivariate-adjusted geometric mean (95% CI) | 3.58 (3.48–3.67) | 3.49 (3.39–3.60) | 3.51 (3.44–3.59) | 3.35 (3.29–3.40) |
| *P*-value | <0.001 | 0.077 | 0.002 |  |
| 2014 fiscal year |  |  |  |  |
| Number of participants | 2 375 | 1 842 | 3 545 | 6 138 |
| Multivariate-adjusted geometric mean (95% CI) | 3.32 (3.24–3.40) | 3.42 (3.33–3.52) | 3.35 (3.28–3.41) | 3.18 (3.14–3.23) |
| *P*-value | 0.02 | <0.001 | <0.001 |  |
| 2015 fiscal year |  |  |  |  |
| Number of participants | 1 448 | 453 | 1 426 | 2 317 |
| Multivariate-adjusted geometric mean (95% CI) | 3.09 (3.00–3.18) | 2.92 (2.77–3.07) | 3.09 (3.00–3.18) | 3.10 (3.03–3.17) |
| *P*-value | 1.000 | 0.214 | 1.000 |  |
|  |  |  |  |  |
| Men (n=10 805) |  |  |  |  |
| 2013 fiscal year |  |  |  |  |
| Number of participants | 563 | 472 | 900 | 1 758 |
| Multivariate-adjusted geometric mean (95% CI) | 3.69 (3.52–3.86) | 3.58 (3.41–3.77) | 3.59 (3.46–3.73) | 3.41 (3.32–3.50) |
| *P*-value | 0.029 | 0.563 | 0.156 |  |
| 2014 fiscal year |  |  |  |  |
| Number of participants | 864 | 681 | 1 331 | 2 212 |
| Multivariate-adjusted geometric mean (95% CI) | 3.45 (3.32–3.59) | 3.52 (3.36–3.68) | 3.60 (3.49–3.72) | 3.29 (3.21–3.37) |
| *P*-value | 0.265 | 0.057 | <0.001 |  |
| 2015 fiscal year |  |  |  |  |
| Number of participants | 531 | 156 | 507 | 830 |
| Multivariate-adjusted geometric mean (95% CI) | 3.24 (3.09–3.40) | 3.16 (2.89–3.45) | 3.26 (3.10–3.42) | 3.31 (3.19–3.44) |
| *P*-value | 1.000 | 1.000 | 1.000 |  |
|  |  |  |  |  |
| Women (n=18 737) |  |  |  |  |
| 2013 fiscal year |  |  |  |  |
| Number of participants | 973 | 809 | 1 460 | 3 063 |
| Multivariate-adjusted geometric mean (95% CI) | 3.51 (3.39–3.63) | 3.45 (3.32–3.58) | 3.47 (3.37–3.57) | 3.31 (3.24–3.37) |
| *P*-value | 0.026 | 0.377 | 0.041 |  |
| 2014 fiscal year |  |  |  |  |
| Number of participants | 1 511 | 1 161 | 2 214 | 3 926 |
| Multivariate-adjusted geometric mean (95% CI) | 3.24 (3.15–3.34) | 3.37 (3.26–3.49) | 3.21 (3.13–3.29) | 3.12 (3.07–3.18) |
| *P*-value | 0.207 | 0.001 | 0.54 |  |
| 2015 fiscal year |  |  |  |  |
| Number of participants | 917 | 297 | 919 | 1 487 |
| Multivariate-adjusted geometric mean (95% CI) | 3.01 (3.12–2.90) | 2.79 (2.62–2.97) | 2.99 (2.89–3.10) | 2.98 (2.90–3.07) |
| *P*-value | 1.000 | 0.355 | 1.000 |  |

Multivariate-adjusted geometric means (95% CI) of the UNa/K ratio were calculated by analysis of covariance with adjustment for age, sex, body mass index, systolic blood pressure, estimated glomerular filtration rate, hemoglobin A1c and low-density lipoprotein cholesterol levels, K6 score, current smoking, current drinking and regular exercise, living alone or not, and having a decreased income or not. Multiple comparisons adjusted by Bonferroni correction were performed.

UNa/K ratio, urine sodium-to-potassium ratio; TC, total collapse; HC, half collapse; PC, partial collapse; ND, no damage; CI, confidence interval

**Supplemental Table 6**. Multivariate-adjusted odds ratios (95% confidence intervals) for high UNa/K ratios according to different periods of investigation

|  | **Extent of house collapse** | | | |
| --- | --- | --- | --- | --- |
|  | **TC** | **HC** | **PC** | **ND** |
| Total (n=29 542) |  |  |  |  |
| 2013 fiscal year |  |  |  |  |
| Number of participants | 1 536 | 1 281 | 2 360 | 4 821 |
| Number of cases with a high UNa/K ratio | 463 | 377 | 693 | 1 198 |
| Multivariate-adjusted OR (95% CI) | 1.30 (1.15–1.48) | 1.26 (1.10–1.44) | 1.26 (1.13–1.40) | Reference |
| *P*-value | <0.001 | 0.001 | <0.001 |  |
| 2014 fiscal year |  |  |  |  |
| Number of participants | 2 375 | 1 842 | 3 545 | 6 138 |
| Number of cases with a high UNa/K ratio | 608 | 509 | 959 | 1 442 |
| Multivariate-adjusted OR (95% CI) | 1.12 (1.01–1.25) | 1.25 (1.11–1.40) | 1.21 (1.10–1.33) | Reference |
| *P*-value | 0.037 | <0.001 | <0.001 |  |
| 2015 fiscal year |  |  |  |  |
| Number of participants | 1 448 | 453 | 1 426 | 2 317 |
| Number of cases with a high UNa/K ratio | 279 | 84 | 312 | 484 |
| Multivariate-adjusted OR (95% CI) | 0.90 (0.77–1.07) | 0.86 (0.67–1.12) | 1.06 (0.90–1.25) | Reference |
| *P*-value | 0.226 | 0.261 | 0.471 |  |
|  |  |  |  |  |
| Men (n=10 805) |  |  |  |  |
| 2013 fiscal year |  |  |  |  |
| Number of participants | 563 | 472 | 900 | 1 758 |
| Number of cases with a high UNa/K ratio | 170 | 142 | 254 | 420 |
| Multivariate-adjusted OR (95% CI) | 1.38 (1.12–1.71) | 1.37 (1.10–1.72) | 1.26 (1.05–1.51) | Reference |
| *P*-value | 0.003 | 0.006 | 0.014 |  |
| 2014 fiscal year |  |  |  |  |
| Number of participants | 864 | 681 | 1 331 | 2 212 |
| Number of cases with a high UNa/K ratio | 223 | 186 | 371 | 510 |
| Multivariate-adjusted OR (95% CI) | 1.16 (0.97–1.39) | 1.25 (1.03–1.52) | 1.29 (1.10–1.50) | Reference |
| *P*-value | 0.111 | 0.024 | 0.001 |  |
| 2015 fiscal year |  |  |  |  |
| Number of participants | 531 | 156 | 507 | 830 |
| Number of cases with a high UNa/K ratio | 98 | 35 | 106 | 191 |
| Multivariate-adjusted OR (95% CI) | 0.76 (0.58–0.99) | 0.97 (0.64–1.46) | 0.88 (0.68–1.16) | Reference |
| *P*-value | 0.045 | 0.875 | 0.369 |  |
|  |  |  |  |  |
| Women (n=18 737) |  |  |  |  |
| 2013 fiscal year |  |  |  |  |
| Number of participants | 973 | 809 | 1 460 | 3 063 |
| Number of cases with a high UNa/K ratio | 293 | 235 | 439 | 778 |
| Multivariate-adjusted OR (95% CI) | 1.26 (1.07–1.48) | 1.20 (1.01–1.42) | 1.26 (1.10–1.45) | Reference |
| *P*-value | 0.005 | 0.039 | 0.001 |  |
| 2014 fiscal year |  |  |  |  |
| Number of participants | 1 511 | 1 161 | 2 214 | 3 926 |
| Number of cases with a high UNa/K ratio | 385 | 323 | 588 | 932 |
| Multivariate-adjusted OR (95% CI) | 1.10 (0.96–1.27) | 1.24 (1.07–1.44) | 1.17 (1.03–1.31) | Reference |
| *P*-value | 0.159 | 0.004 | 0.012 |  |
| 2015 fiscal year |  |  |  |  |
| Number of participants | 917 | 297 | 919 | 1 487 |
| Number of cases with a high UNa/K ratio | 181 | 49 | 206 | 293 |
| Multivariate-adjusted OR (95% CI) | 1.00 (0.82–1.23) | 0.81 (0.58–1.12) | 1.18 (0.96–1.44) | Reference |
| *P*-value | 0.984 | 0.201 | 0.111 |  |

Multivariate-adjusted odds ratios (ORs) for high UNa/K ratios were calculated using a logistic regression model with adjustment for age, sex, body mass index, systolic blood pressure, estimated glomerular filtration rate, hemoglobin A1c and low-density lipoprotein cholesterol levels, K6 score, current smoking, current drinking and regular exercise, living alone or not, and having a decreased income or not. The cutoff was the 75th percentile in UNa/K ratio for each sex in the study group. The UNa/K ratio in the 75th percentile or higher was defined as “high UNa/K ratio.” The 75th percentile in UNa/K ratio was 5.09% for men and 4.76% for women.

UNa/K ratio, urine sodium-to-potassium ratio; TC, total collapse; HC, half collapse; PC, partial collapse; ND, no damage; OR, odds ratio; CI, confidence interval

**Supplemental Table 7**. Multivariate-adjusted geometric means (95% confidence intervals) of the UNa/K ratio according to different age groups

|  | **Extent of house collapse** | | | |
| --- | --- | --- | --- | --- |
|  | **TC** | **HC** | **PC** | **ND** |
| Total (n=29 542) |  |  |  |  |
| Age, 20–49 years |  |  |  |  |
| Number of participants | 983 | 567 | 1 138 | 2 345 |
| Multivariate-adjusted geometric mean (95% CI) | 3.59 (3.46–3.73) | 3.42 (3.26–3.59) | 3.22 (3.11–3.33) | 3.45 (3.37–3.53) |
| *P*-value | 0.417 | 1.000 | 0.007 |  |
| Age, 50–64 years |  |  |  |  |
| Number of participants | 1 953 | 1 392 | 2 756 | 4 773 |
| Multivariate-adjusted geometric mean (95% CI) | 3.29 (3.21–3.37) | 3.37 (3.27–3.47) | 3.38 (3.31–3.45) | 3.23 (3.18–3.28) |
| *P*-value | 1.000 | 0.094 | 0.004 |  |
| Age, 65–74 years |  |  |  |  |
| Number of participants | 2 423 | 1 617 | 3 437 | 6 157 |
| Multivariate-adjusted geometric mean (95% CI) | 3.25 (3.18–3.33) | 3.37 (3.28–3.47) | 3.36 (3.30–3.43) | 3.14 (3.10–3.19) |
| *P*-value | 0.014 | <0.001 | <0.001 |  |
|  |  |  |  |  |
| Men (n=10 805) |  |  |  |  |
| Age, 20–49 years |  |  |  |  |
| Number of participants | 300 | 166 | 286 | 613 |
| Multivariate-adjusted geometric mean (95% CI) | 3.68 (3.45–3.92) | 3.56 (3.26–3.88) | 3.36 (3.15–3.59) | 3.63 (3.46–3.79) |
| *P*-value | 1.000 | 1.000 | 0.412 |  |
| Age, 50–64 years |  |  |  |  |
| Number of participants | 646 | 426 | 863 | 1 500 |
| Multivariate-adjusted geometric mean (95% CI) | 3.48 (3.33–3.64) | 3.58 (3.38–3.78) | 3.63 (3.50–3.78) | 3.38 (3.28–3.48) |
| *P*-value | 1.000 | 0.462 | 0.020 |  |
| Age, 65–74 years |  |  |  |  |
| Number of participants | 1 012 | 717 | 1 589 | 2 687 |
| Multivariate-adjusted geometric mean (95% CI) | 3.40 (3.28–3.52) | 3.43 (3.29–3.58) | 3.50 (3.41–3.60) | 3.25 (3.18–3.32) |
| *P*-value | 0.217 | 0.160 | <0.001 |  |
|  |  |  |  |  |
| Women (n=18 737) |  |  |  |  |
| Age, 20–49 years |  |  |  |  |
| Number of participants | 683 | 401 | 852 | 1 732 |
| Multivariate-adjusted geometric mean (95% CI) | 3.57 (3.41–3.73) | 3.37 (3.18–3.57) | 3.17 (3.04–3.30) | 3.38 (3.29–3.48) |
| *P*-value | 0.314 | 1.000 | 0.051 |  |
| Age, 50–64 years |  |  |  |  |
| Number of participants | 1 307 | 966 | 1 893 | 3 273 |
| Multivariate-adjusted geometric mean (95% CI) | 3.20 (3.11–3.30) | 3.27 (3.16–3.39) | 3.27 (3.19–3.35) | 3.16 (3.11–3.23) |
| *P*-value | 1.000 | 0.564 | 0.233 |  |
| Age, 65–74 years |  |  |  |  |
| Number of participants | 1 411 | 900 | 1 848 | 3 470 |
| Multivariate-adjusted geometric mean (95% CI) | 3.14 (3.05–3.24) | 3.33 (3.21–3.46) | 3.26 (3.18–3.34) | 3.06 (3.00–3.12) |
| *P*-value | 0.866 | <0.001 | 0.001 |  |

Multivariate-adjusted geometric means (95% CI) of the UNa/K ratio were calculated by analysis of covariance with adjustment for age, sex, body mass index, systolic blood pressure, estimated glomerular filtration rate, hemoglobin A1c and low-density lipoprotein cholesterol levels, K6 score, current smoking, current drinking and regular exercise, living alone or not, and having a decreased income or not. Multiple comparisons adjusted by Bonferroni correction were performed.

UNa/K ratio, urine sodium-to-potassium ratio; TC, total collapse; HC, half collapse; PC, partial collapse; ND, no damage; CI, confidence interval

**Supplemental Table 8**. Multivariate-adjusted odds ratios (95% confidence intervals) for high UNa/K ratios according to different age groups

|  | **Extent of house collapse** | | | |
| --- | --- | --- | --- | --- |
|  | **TC** | **HC** | **PC** | **ND** |
| Total (n=29 542) |  |  |  |  |
| Age, 20–49 years |  |  |  |  |
| Number of participants | 983 | 567 | 1 138 | 2 345 |
| Number of cases with a high UNa/K ratio | 310 | 163 | 283 | 673 |
| Multivariate-adjusted OR (95% CI) | 1.15 (0.98–1.35) | 1.004 (0.82–1.23) | 0.82 (0.70–0.97) | Reference |
| *P*-value | 0.098 | 0.973 | 0.017 |  |
| Age, 50–64 years |  |  |  |  |
| Number of participants | 1 953 | 1 392 | 2 756 | 4 773 |
| Number of cases with a high UNa/K ratio | 496 | 370 | 795 | 1 085 |
| Multivariate-adjusted OR (95% CI) | 1.16 (1.02–1.31) | 1.23 (1.07–1.41) | 1.38 (1.24–1.53) | Reference |
| *P*-value | 0.020 | 0.003 | <0.001 |  |
| Age, 65–74 years |  |  |  |  |
| Number of participants | 2 423 | 1 617 | 3 437 | 6 157 |
| Number of cases with a high UNa/K ratio | 544 | 437 | 886 | 1 365 |
| Multivariate-adjusted OR (95% CI) | 1.02 (0.91–1.14) | 1.30 (1.15–1.47) | 1.22 (1.11–1.34) | Reference |
| *P*-value | 0.779 | <0.001 | <0.002 |  |
|  |  |  |  |  |
| Men (n=10 805) |  |  |  |  |
| Age, 20–49 years |  |  |  |  |
| Number of participants | 300 | 166 | 286 | 613 |
| Number of cases with a high UNa/K ratio | 91 | 49 | 68 | 174 |
| Multivariate-adjusted OR (95% CI) | 1.10 (0.81–1.49) | 1.06 (0.73–1.54) | 0.79 (0.57–1.09) | Reference |
| *P*-value | 0.542 | 0.775 | 0.147 |  |
| Age, 50–64 years |  |  |  |  |
| Number of participants | 646 | 426 | 863 | 1 500 |
| Number of cases with a high UNa/K ratio | 168 | 117 | 258 | 357 |
| Multivariate-adjusted OR (95% CI) | 1.13 (0.91–1.39) | 1.21 (0.95–1.55) | 1.37 (1.13–1.65) | Reference |
| *P*-value | 0.276 | 0.122 | 0.001 |  |
| Age, 65–74 years |  |  |  |  |
| Number of participants | 1 012 | 717 | 1 589 | 2 687 |
| Number of cases with a high UNa/K ratio | 232 | 197 | 405 | 590 |
| Multivariate-adjusted OR (95% CI) | 1.06 (0.89–1.26) | 1.35 (1.12–1.63) | 1.22 (1.05–1.41) | Reference |
| *P*-value | 0.528 | 0.002 | 0.008 |  |
|  |  |  |  |  |
| Women (n=18 737) |  |  |  |  |
| Age, 20–49 years |  |  |  |  |
| Number of participants | 683 | 401 | 852 | 1 732 |
| Number of cases with a high UNa/K ratio | 219 | 114 | 215 | 499 |
| Multivariate-adjusted OR (95% CI) | 1.17 (0.96–1.41) | 0.98 (0.77–1.25) | 0.83 (0.69–1.01) | Reference |
| *P*-value | 0.115 | 0.879 | 0.056 |  |
| Age, 50–64 years |  |  |  |  |
| Number of participants | 1 307 | 966 | 1 893 | 3 273 |
| Number of cases with a high UNa/K ratio | 328 | 253 | 537 | 728 |
| Multivariate-adjusted OR (95% CI) | 1.17 (1.01–1.36) | 1.24 (1.05–1.46) | 1.38 (1.22–1.58) | Reference |
| *P*-value | 0.039 | 0.011 | <0.001 |  |
| Age, 65–74 years |  |  |  |  |
| Number of participants | 1 411 | 900 | 1 848 | 3 470 |
| Number of cases with a high UNa/K ratio | 312 | 240 | 481 | 775 |
| Multivariate-adjusted OR (95% CI) | 0.99 (0.85–1.15) | 1.27 (1.09–1.50) | 1.22 (1.07–1.40) | Reference |
| *P*-value | 0.866 | 0.006 | 0.003 |  |

Multivariate-adjusted odds ratios (ORs) for high UNa/K ratios were calculated using a logistic regression model with adjustment for age, sex, body mass index, systolic blood pressure, estimated glomerular filtration rate, hemoglobin A1c and low-density lipoprotein cholesterol levels, K6 score, current smoking, current drinking and regular exercise, living alone or not, and having a decreased income or not. The cutoff was the 75th percentile in UNa/K ratio for each sex in the study group. The UNa/K ratio in the 75th percentile or higher was defined as “high UNa/K ratio.” The 75th percentile in UNa/K ratio was 5.09% for men and 4.76% for women.

UNa/K ratio, urine sodium-to-potassium ratio; TC, total collapse; HC, half collapse; PC, partial collapse; ND, no damage; OR, odds ratio; CI, confidence interval

**Supplementary Figures**

**
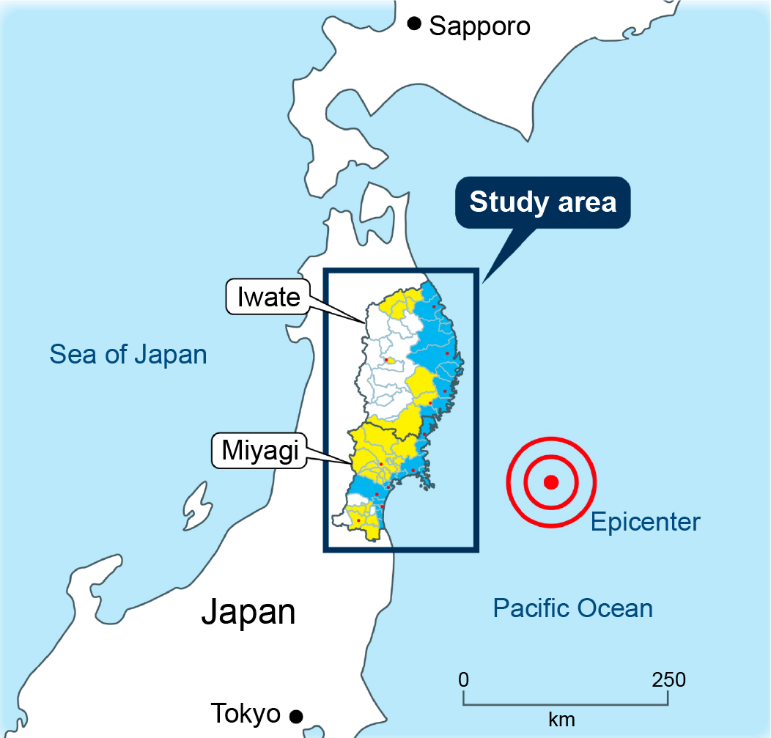
**

**Supplemental Figure 1**. Locations of Iwate and Miyagi Prefectures and the epicenter of the 2011 Great East Japan Earthquake. Colored cities indicate study areas of the Tohoku Medical Megabank Project Community-Based Cohort Study. Blue-colored cities are coastal cities, and yellow ones are inland. The epicenter of the earthquake is marked with a red dot.


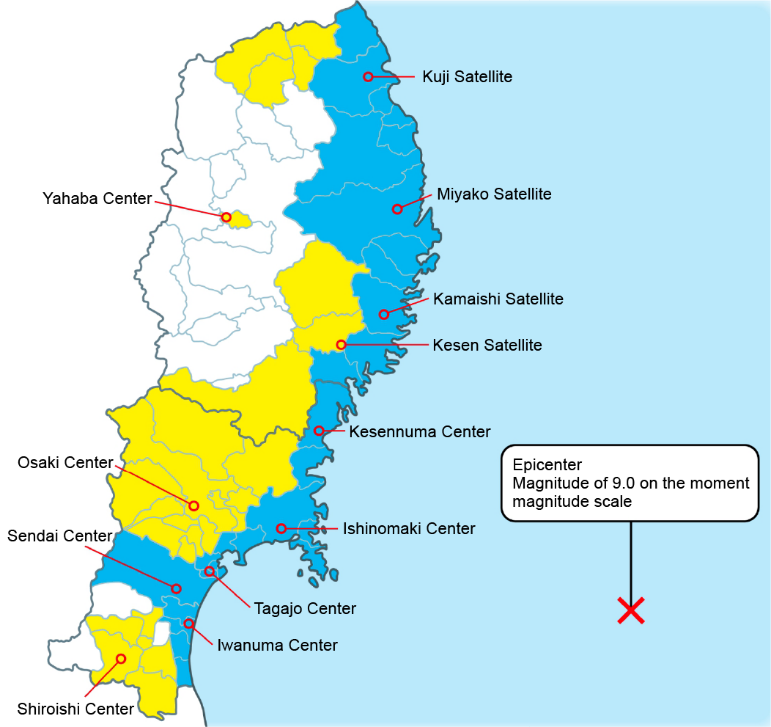


**Supplemental Figure 2**. Municipalities of Miyagi and Iwate Prefectures. Red circles indicate community support centers in Miyagi Prefecture and satellites in Iwate Prefecture.
